# Supplementary material for: Empirical methods for controlling false positives and estimating confidence in ChIP-Seq peaks
Source: BMC Bioinformatics. 2008 Dec 5;9:523. doi: 10.1186/1471-2105-9-523 (PMC2628906; doi:10.1186/1471-2105-9-523)
Supplement: Additional File 1 — A variety of html documents from the USeq web site detailing the available applications, their best usage, output file type descriptions, command line menus, etc. [file 1471-2105-9-523-S1.zip › USeqUserGuides/JavaDoc.zip!/JavaDoc/trans/roc/SgrBacCounter.html]

SgrBacCounter


|  |  |  |
| --- | --- | --- |
| **Overview** \| **Package** \| **Class** \| **Use** \| **Tree** \| **Deprecated** \| **Index** \| **Help** | |  |
| **PREV CLASS**   NEXT CLASS | **FRAMES**    **NO FRAMES**     **All Classes** |  |
| SUMMARY: NESTED \| FIELD \| CONSTR \| METHOD | DETAIL: FIELD \| CONSTR \| METHOD |  |


---


## trans.roc Class SgrBacCounter

```
java.lang.Object
  trans.roc.SgrBacCounter
```

---

``` public class SgrBacCounter extends java.lang.Object ```

Application for scoring '.sgr' files for positives and negatives in relation to the BAC spike in data.

---

**Constructor Summary**

`SgrBacCounter(java.lang.String[] args)`


| **Method Summary** | |
| --- | --- |
| `Bin` | `findBin(Sgr sgr)`             Finds an appropriate bin given an sgr object |
| `static double` | `fractionPositive(java.util.ArrayList doubles, double scoreCutOff, double divider)`             Given an ArrayList of Double, calculates the fraction that is >= scoreCutOff. |
| `static void` | `main(java.lang.String[] args)` |
| `void` | `makeBins()` |
| `void` | `makePositives()` |
| `static int` | `positive(Positive[] pos, Sgr sgr)`             Checks to see if an sgr object is one of the positives, if not returns -1, otherwise returns index number. |
| `void` | `scanSgrArray()`             Scans an sgr array, sorting into bins. |

**Methods inherited from class java.lang.Object**

`equals, getClass, hashCode, notify, notifyAll, toString, wait, wait, wait`

**Constructor Detail**

### SgrBacCounter

```
public SgrBacCounter(java.lang.String[] args)
```


**Method Detail**

### scanSgrArray

```
public void scanSgrArray()
```

:   Scans an sgr array, sorting into bins.

---


### positive

```
public static int positive(Positive[] pos,
                           Sgr sgr)
```

:   Checks to see if an sgr object is one of the positives, if not returns -1, otherwise returns index number.

---


### findBin

```
public Bin findBin(Sgr sgr)
```

:   Finds an appropriate bin given an sgr object

---


### main

```
public static void main(java.lang.String[] args)
```

---


### fractionPositive

```
public static double fractionPositive(java.util.ArrayList doubles,
                                      double scoreCutOff,
                                      double divider)
```

:   Given an ArrayList of Double, calculates the fraction that is >= scoreCutOff.

---


### makeBins

```
public void makeBins()
```

---


### makePositives

```
public void makePositives()
```


---


|  |  |  |
| --- | --- | --- |
| **Overview** \| **Package** \| **Class** \| **Use** \| **Tree** \| **Deprecated** \| **Index** \| **Help** | |  |
| **PREV CLASS**   NEXT CLASS | **FRAMES**    **NO FRAMES**     **All Classes** |  |
| SUMMARY: NESTED \| FIELD \| CONSTR \| METHOD | DETAIL: FIELD \| CONSTR \| METHOD |  |


---
